# Supplementary material for: Measuring Variant-Specific Neutralizing Antibody Profiles after Bivalent SARS-CoV-2 Vaccinations Using a Multivariant Surrogate Virus Neutralization Microarray
Source: Vaccines (Basel). 2024 Jan 18;12(1):94. doi: 10.3390/vaccines12010094 (PMC10818493; doi:10.3390/vaccines12010094)
Supplement: Supplementary file 1 [file vaccines-12-00094-s001.zip › vaccines-2782435-supplementary.pdf]

Supplementary information

Supplementary Table S1: Cohort characteristics

Cohort	n	Female (n)	Age (median)	Age (min)	Age (max)	Days since last vacc./inf. (median)	Days since last vacc./inf. (min)	Days since last vacc./inf. (max)
**2 x Vacc.-monov.**	11	9	40	20	81	20	15	34
**3 x Vacc.-monov.**	14	11	45.5	27	64	27.5	15	40
**4 x Vacc.-biv. (BA.1/WT)**	9	4	52	38	62	21	20	28
**4 x Vacc.-biv. (BA.5/WT)**	13	8	50	32	57	21	21	30
**WT infection**	18	4	48	22	77	19.5	11	25
**Prepandemic control**	30	15	23	19	55	/	/	/

Age in years, Days since last vacc./inf.: Days since last vaccination (vaccinated cohorts) or infection (WT infection cohort). /: not applicable.

Supplementary Table S2: Vaccination history

Cohort	Vaccination history	n
**2 x Vacc.-monov.**	P + P	10
	P + P	1
**3 x Vacc.-monov.**	P + P + P	11
	P + P + M	3
**4 x Vacc.-biv. (BA.1/WT)**	P + P + P + P-BA.1	8
	AZ + AZ + P + P-BA.1	1
**4 x Vacc.-biv. (BA.5/WT)**	P + P + P + P-BA.5	9
	J + P + P + P-BA.5	3
	AZ + AZ + P + P-BA.5	1
**WT infection**	not vaccinated	18
**Prepandemic control**	not vaccinated	30

AZ: Astra-Zeneca "Vaxzevria" ChAdOx1; P: Monovalent Biontech/Pfizer "Comirnaty" BNT162b2; P-BA.1: Biontech/Pfizer Bivalent (WT/BA.1) BNT162b2 BA.1; P-BA.5: Biontech/Pfizer Bivalent (WT/BA.5) BNT162b2 BA.5, M: Moderna "Spikevax" mRNA-1273. n: number.

Supplementary Table S3: sVNT cutoff values obtained by ROC and Youden's Index

	Cutoff value	Sensitivity	Specificity
**WT**	26	>99 %	>99 %
**Delta**	43	>99 %	>99 %
**BA.1**	24	92 %	78 %
**BA.2**	9	91 %	87 %
**BA.5**	28	88 %	92 %

Supplementary Table S4: Pairwise cohortwise-comparisons of variant/WT ratios (Wilcoxon test)

Ratio	Cohort	WT infection	2 x Vacc.-monov.	3 x Vacc.-monov.	4 x Vacc.-biv. (BA.1/WT)
**Delta/WT**	**2 x Vacc.-monov.**	n.s.			
	**3 x Vacc.-monov.**	***	****		
	**4 x Vacc.-biv. (BA.1/WT)**	**	***	n.s.	
	**4 x Vacc.-biv. (BA.5/WT)**	**	***	n.s.	n.s.
**BA.1/WT**	**2 x Vacc.-monov.**	**			
	**3 x Vacc.-monov.**	n.s.	n.s.		
	**4 x Vacc.-biv. (BA.1/WT)**	n.s.	***	*	
	**4 x Vacc.-biv. (BA.5/WT)**	*	****	**	n.s.
**BA.2/WT**	**2 x Vacc.-monov.**	n.s.			
	**3 x Vacc.-monov.**	****	****		
	**4 x Vacc.-biv. (BA.1/WT)**	****	****	n.s.	
	**4 x Vacc.-biv. (BA.5/WT)**	****	****	n.s.	n.s.
**BA.5/WT**	**2 x Vacc.-monov.**	n.s.			
	**3 x Vacc.-monov.**	n.s.	n.s.		
	**4 x Vacc.-biv. (BA.1/WT)**	n.s.	n.s.	n.s.	
	**4 x Vacc.-biv. (BA.5/WT)**	***	***	**	n.s.

**** $p < 0.0001$, *** $p < 0.001$, ** $p < 0.01$, * $p < 0.05$, n.s.: not significant ($p > 0.05$) in pairwise Wilcoxon signed rank tests comparing the variant / WT ratios between cohorts for each variant (multiplicity adjusted using Bonferroni-Holm within each variant).

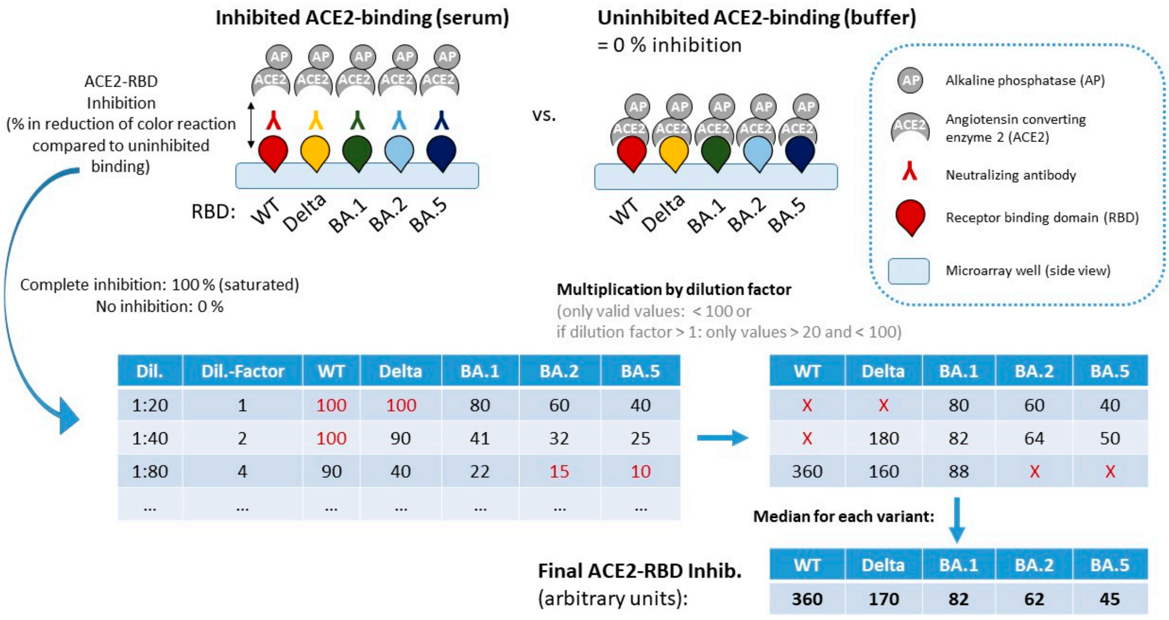

Supplementary Figure S1: Schematic overview of the sVNT result calculation

ACE2-RBD inhibiton was obtained by measuring the reduction in binding of ACE2-AP to recombinant variant RBDs in the presence/absence of serum neutralizing antibodies relative to the control well (buffer). A value of 100 indicated total inhibiton (i.e. no binding and no color reaction). This was repeated at serial two-fold diluations of 1:20 up to 1:360 for each serum, and, additionally up to 1:2560 in some cases. Next, all valid values (i.e. not oversaturated (= 100) for all dilutions and not below 20 for all dilutions except 1:20) were corrected for the dilution relative to 1:20 (i.e. multiplication by the dilution factor relative to 1:20). Invalid values are indicated in red font. Finally, the median of all valid values was calculated for each variant. Due to the dilution correction, values above 100 can occur; thus, the results are given in arbitrary units instead of % inhibition. ACE2-AP: Angiotensin converting enzyme 2 bound to alkaline phosphate, Dil.: dilution; Dil.-Factor: dilution factor; RBD: receptor binding domain, WT: wildtype.

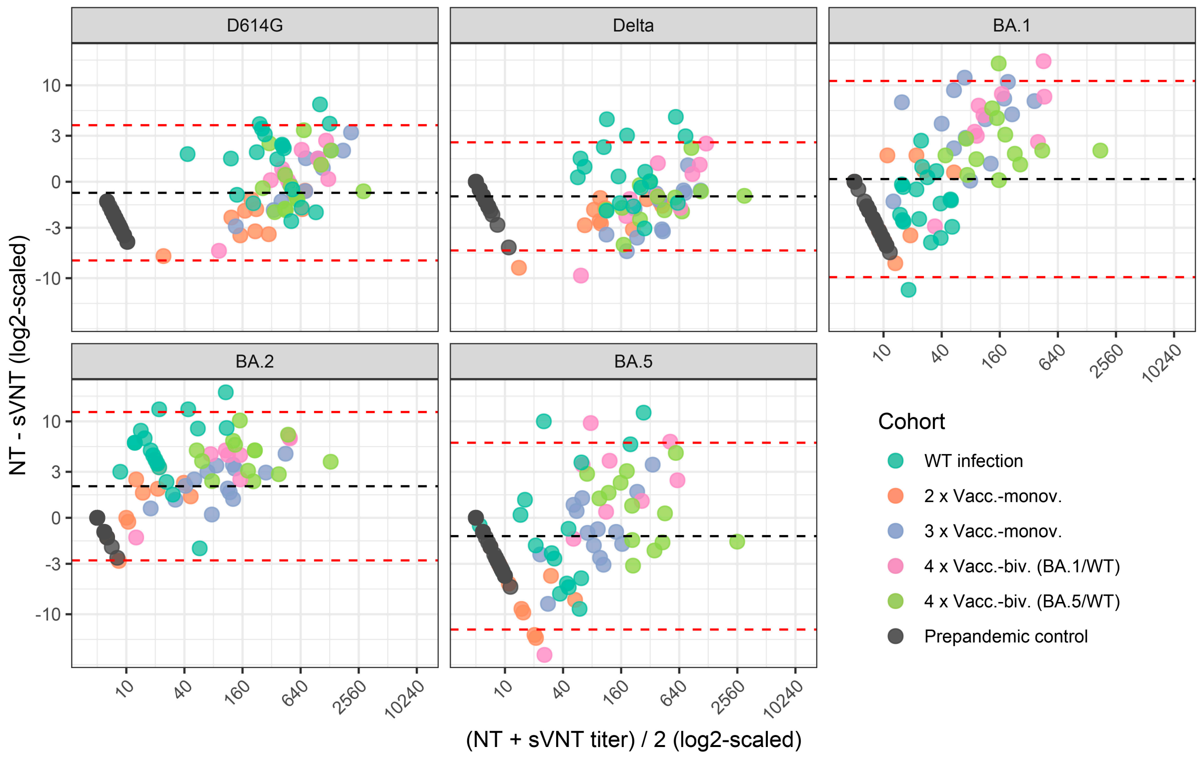

Supplementary Figure S2: Bland-Altman analysis

The panels display the virus variants against which neutralizing activity was measured using a sVNT and NT. The x-axis shows the mean result ("titer") of the two measurement methods (mean calculated after log2-transformation). The y-axis shows the difference between the two measurement methods (also calculated after log2-transformation). Each serum is represented by one dot (color coded for the cohorts). The black dotted line indicates the mean and the red lines indicate the 95% agreement intervals. biv.: bivalent; NT: neutralization test; sVNT: surrogate virus neutralization test; Vacc.: vaccinated; WT: wildtype.
